# Effect of 4-Phenylbutyric Acid and Tauroursodeoxycholic Acid on Magnesium and Calcium Metabolism in Streptozocin-Induced Type 1 Diabetic Mice

**DOI:** 10.1007/s12011-018-1494-8

**Published:** 2018-08-31

**Authors:** Qi Zhou, Wenjia Guo, Yanan Jia, Jiancheng Xu

**Affiliations:** 1 Department of Pediatrics, First Hospital of Jilin University, Changchun, 130021 China; 2 Department of Laboratory Medicine, First Hospital of Jilin University, 71 Xinmin Street, Changchun, 130021 China

**Keywords:** Magnesium, Calcium, Diabetes, 4-Phenylbutyric acid, Tauroursodeoxycholic acid

## Abstract

Recent evidence has identified a role of micronutrients, such as magnesium (Mg^2+^) and calcium (Ca^2+^), in glycemic control. 4-Phenylbutyric acid (PBA) and tauroursodeoxycholic acid (TUDCA) are molecular chaperones that can improve protein folding and alleviate endoplasmic reticulum (ER) stress. Increasingly, research is focusing on the association between molecular chaperones and micronutrients. This study established and characterized a mouse model of type 1 diabetes (T1D) and investigated the effect of PBA and TUDCA on Mg^2+^ and Ca^2+^ metabolism in these mice. T1D was established in Friend virus B-type mice using multiple low doses of streptozotocin. Mice were administered chaperones. Mg^2+^and Ca^2+^ levels in tissues and serum were detected using acid digestion and ICP-MS. At 2 weeks and 2 months after chaperone administration was initiated, Mg^2+^ levels in the heart, liver, kidney, and serum and Ca^2+^ levels in spleen and serum of T1D mice were significantly decreased compared with controls; Ca^2+^ levels in the kidney and muscle of T1D mice were significantly increased; Mg^2+^ and Ca^2+^ levels in the heart, liver, kidney, muscle, spleen, and serum were positively correlated in control and T1D mice; and PBA restored renal Mg^2+^ levels to normal values and TUDCA restored hepatic, renal, and serum Mg^2+^ levels and renal and serum Ca^2+^ levels to normal values in T1D mice. PBA restored muscular Ca^2+^ levels to normal values in T1D mice at 2 months after chaperone or vehicle administration was initiated. Further research is required to investigate the underlying mechanisms by which chaperones regulate micronutrients in diabetes.

## Introduction

Globally, non-communicable diseases (NCDs), including cardiovascular disease, diabetes mellitus, cancer, and chronic respiratory diseases [1], are associated with substantial morbidity and mortality. In particular, diabetes is a growing public health concern, with prevalence increasing in developing countries [2, 3]. Diabetes can cause complications in multiple organs, manifesting as diabetic retinopathy, neuropathy, and nephropathy and causing disability and mortality in many individuals [4]. Type 1 diabetes (T1D) is characterized by hyperglycemia resulting from autoimmune destruction of insulin-secreting pancreatic β-islet cells.

Nutrition is a critical component of diabetes management. Previously, interest in the role of nutrients in diabetes has focused on macronutrients, including carbohydrate and fat. More recent evidence has identified a role for micronutrients, such as magnesium (Mg^2+^) [5] and calcium (Ca^2+^) [6], in glycemic control. Mg^2+^ is the most abundant divalent intracellular cation, the second most abundant intracellular ion, and the fourth most abundant mineral in the human body [7]. Some studies show that Mg^2+^ may improve insulin sensitivity and prevent diabetes [8]; however, two cohort studies (13,076 persons/year) indicated that low Mg^2+^ intake was not a risk factor for diabetes [9], and six randomized controlled trials demonstrated that Mg^2+^ was not effective for glycemic control in patients with type 2 diabetes (T2D) [9]. Ca^2+^ is the most abundant ion in the body. Alterations in Ca^2+^ homeostasis are associated with organelle dysfunction and stress responses in metabolic organs such as liver and adipose tissue [6]. Both T1D and T2D are characterized by changes in Ca^2+^ and bone metabolism, in part due to impaired Ca^2+^ absorption from intestine [10]. According to our previous studies [11, 12], diabetes induces endoplasmic reticulum stress (ERS) and vice versa; the effect on trace elements, especially on Ca^2+^, is quite clear.

Chemical or drug chaperones, including 4-phenylbutyric acid (PBA) and tauroursodeoxycholic acid (TUDCA), can improve protein folding and lessen ERS. Increasingly, research is focusing on the association between molecular chaperones and micronutrients. In a mouse model of chronic kidney disease, short-term treatment with TUDCA normalized Ca^2+^ content in vascular smooth muscle, providing a potential strategy for therapeutic management of vascular calcification [13]. In HeLa human cervical cancer cells, propofol-induced disruption of intracellular Ca^2+^ balance was inhibited by TUDCA [14]. In vascular smooth muscle cells, the Runx2 expression, alkaline phosphatase activity, and Ca^2+^ nodules induced by high concentrations of glucose were decreased by PBA pretreatment [15]. In a rat model of severe burn injury, PBA treatment prevented swelling of ER, changed the expression of ERS markers and Ca^2+^ release, and reduced calpain activation and skeletal muscle damage/wasting [16].

Currently, reports on effects of PBA or TUDCA on Mg^2+^ and Ca^2+^ metabolism in diabetes are scarce. This study established and characterized a mouse model of T1D and investigated the effect of PBA and TUDCA on Mg^2+^ and Ca^2+^ metabolism in these mice.

## Materials and Methods

### Animal Models

Eight-week-old, male Friend virus B-type (FVB) mice purchased from Vital River Laboratories (Beijing, China) were housed at the Experimental Animal Center in the College of Basic Medical Sciences, Jilin University. Mice were maintained at 22 °C with a 12-h light/dark cycle and were provided free access to standard rodent chow and water. This study was approved by the institutional ethics committee of the First Hospital of Jilin University.

Mice were randomly divided into six groups: non-diabetic control mice (CON); diabetic mice (DM); diabetic mice treated with PBA (PBA + DM); diabetic mice treated with TUDCA (TUDCA + DM); non-diabetic control mice treated with PBA (PBA); and non-diabetic control mice treated with TUDCA (TUDCA). Mice in all groups were fed with standard laboratory chow (Mouse Feed Food, no. 8061, Chengdu Dashuo Laboratory Animal Co., Ltd., Chengdu, China), which contained 18% protein, 4% fat, 5% fiber, 10% ash content, and 1.4% Ca.

T1D was induced in mice with streptozotocin (STZ) (Sigma Chemical Co., St. Louis, MO, USA) dissolved in sodium citrate buffer (pH 4.5) administered intraperitoneally at 40 mg/kg body weight daily for 5 days (multiple low-dose STZ [MLD-STZ] model). After the very last injection of STZ on the 5th day, blood glucose obtained from the tail-vein was measured using a Glucometer (Bayer HealthCare, Mishawaka, IN, USA). STZ-treated mice with fasting blood glucose > 12 mmol/L were considered diabetic [11].

Diabetic mice were acclimatized to PBA or TUDCA by pretreatment with 100 μL PBA (Merck KGaA, Hohenbrunn, Germany) in drinking water daily or 100 μL TUDCA (Calbiochem, La Jolla, CA, USA) by intraperitoneal injection twice each day (8 am and 8 pm). After 3 days, at 8 am, fasting blood glucose obtained from the tail-vein was measured, and chaperone or vehicle administration was initiated (time = day 0). PBA was administered by gavage (500 mg/kg at 8 am and 8 pm, total 1 g/kg/day), and TUDCA was administered intraperitoneally (250 mg/kg at 8 am and 8 pm, total 500 mg/kg/day), in divided doses daily. Controls for PBA- or TUDCA-treated diabetic mice were administered the same volume of vehicle by gavage or intraperitoneal injection, respectively. Fasting blood glucose and body weight were monitored regularly.

Mice were sacrificed at 2 weeks and 2 months after treatment initiation with PBA or TUDCA using 2% sodium pentobarbital (30 mg/kg, intraperitoneal) and cardiac puncture. The heart, liver, kidney, spleen, and soleus muscles were removed and stored at − 80 °C before processing. Blood was collected into metal-free tubes for analysis of biochemical parameters or Mg^2+^ and Ca^2+^ levels, respectively. Blood was centrifuged at 12000 r/min and 4 °C for 5 min. Serum was aliquoted into Eppendorf tubes soaked in 10% (*w*/*w*) nitric acid, shock frozen, and stored at − 80 °C until further analysis.

### Determination of Mg^2+^ and Ca ^2+^ by Acid Digestion Followed by ICP-MS

Preweighed samples of the heart, liver, kidney, soleus muscle, and spleen were digested in 5 mL nitric acid at room temperature for 12 h and at 110 °C for 8 h. 0.5 mL serum were digested in 2 mL 10% nitric acid at room temperature for 30 min. Subsequently, serum was digested at 100 °C for 2 h. Deionized water was added to each sample to increase the volume to 15 mL. Mg^2+^ and Ca^2+^ levels were assessed by Agilent Technologies 7700 Series ICP-MS equipment (Agilent Technologies, Santa Clara, CA, USA). Rf Power was 1550 W, nebulizer gas flow rate was maintained at 1.05 L/min, and a sample-specific heating program was applied. Standard curves were plotted, and Mg^2+^ and Ca^2+^ levels were calculated as micrograms per liter wet tissue.

### Other Measurements

Glycosylated serum protein (GSP), blood urea nitrogen (BUN), creatinine (Cre), uric acid (UA), total cholesterol (CHO), triglyceride (TG), high-density lipoprotein (HLDL), and low-density lipoprotein cholesterol (LDL) levels were measured using a Hitachi 7600-010 Clinical Chemistry Analyzer (Hitachi, Tokyo, Japan) with the manufacturer’s reagents, calibrators, and quality control (QC) products (Liquid Assay Multiqual Controls level 1, level 2, level 3 by Bio-Rad Laboratories, Inc.).

### Statistical Analysis

Statistical analyses were performed using SPSS v21.0. Continuous variables are presented as mean ± SE. Between-group comparisons were evaluated with Student’s *t* test. Associations between serum Mg^2+^ and Ca^2+^ levels as a continuous variable and biochemical parameters were assessed with Spearman’s rank correlation analysis. All reported *p* values are two-sided, and *p* < 0.05 was considered significant.

## Results

### Characteristics of Mice

Characteristics of mice sacrificed after 2 weeks and 2 months of treatment with PBA or TUDCA are summarized in Table 1. At 2 weeks and 2 months, the DM mice had a significantly lower body weight (2 weeks, 23.6 ± 0.78 vs. 28.7 ± 0.23 g; 2 months, 23.1 ± 0.25 vs. 33.4 ± 0.62 g; *P* < 0.05) and higher fasting blood glucose (2 weeks, 31.9 ± 0.64 vs. 7.2 ± 0.45 mmol/L; 2 months, 33.7 ± 0.61 vs. 8.8 ± 0.24 mmol/L; *P* < 0.05) than the control mice. At 2 weeks and 2 months, PBA + DM or TUDCA + DM mice had significantly higher body weight (2 weeks, PBA + DM 28.8 ± 0.49 g, TUDCA + DM 26.0 ± 0.35 g; 2 months, PBA + DM 25.8 ± 0.28 g, TUDCA + DM 26.1 ± 0.20 g; *P* < 0.05 vs. DM mice) and significantly lower blood glucose (2 weeks, PBA + DM 26.4 ± 0.47 mmol/L, TUDCA + DM 21.4 ± 0.84 mmol/L; 2 months, PBA + DM 28.5 ± 0.76 mmol/L, TUDCA + DM 23.1 ± 0.71 mmol/L; *P* < 0.05 vs. DM mice) than the DM mice, although most values did not return to control levels.

**Table 1:** Characteristics of the DM mice after 2 weeks and 2 months of treatment with PBA or TUDCA

	2 weeks					2 months						
	CON	PBA	TUDCA	PBA + DM	TUDCA + DM	DM	CON	PBA	TUDCA	PBA + DM	TUDCA + DM	DM
BW (g)	28.7 ± 0.23	29.1 ± 0.46	28.9 ± 0.30	28.8 ± 0.49^#^	26.0 ± 0.35**^#^	23.6 ± 0.78*	33.4 ± 0.62	33.6 ± 0.33	33.7 ± 0.53	25.8 ± 0.28***^#^	26.1 ± 0.20**^#^	23.1 ± 0.25*
GLU (mmol/L)	7.2 ± 0.45	7.2 ± 0.42	7.2 ± 0.39	26.4 ± 0.47***^#^	21.4 ± 0.84**^#^	31.9 ± 0.64*	8.8 ± 0.24	8.9 ± 0.13	8.6 ± 0.21	28.5 ± 0.76***^#^	23.1 ± 0.71**^#^	33.7 ± 0.61*
GSP (μmol/L)	–	–	–	–	–	–	259.6 ± 17.9	260.3 ± 10.2	261.1 ± 9.4	460.6 ± 11.5***	431.7 ± 11.9**	475.4 ± 19.2*
BUN (mmol/L)	–	–	–	–	–	–	11.3 ± 0.39	11.3 ± 0.32	11.5 ± 0.40	16.1 ± 0.31***	15.3 ± 0.55**	16.8 ± 0.59*
Cre (mmol/L)	–	–	–	–	–	–	12.7 ± 0.28	12.3 ± 0.28	12.2 ± 0.24	13.3 ± 0.94^#^	18.4 ± 0.84**	20.5 ± 2.30*
UA (μmol/L)	–	–	–	–	–	–	133.9 ± 2.1	133.6 ± 2.2	134.3 ± 2.5	130.0 ± 5.2	140.9 ± 3.4	134.3 ± 8.6
Mg (mg/L)	38.9 ± 0.95	39.5 ± 1.03	39.8 ± 1.16	34.5 ± 1.10***	40.9 ± 0.79^#^	33.5 ± 0.89*	38.7 ± 1.75	38.4 ± 0.47	38.7 ± 1.61	35.4 ± 0.86***	41.3 ± 1.39^#^	33.7 ± 0.30*
Ca (mg/L)	111.3 ± 3.4	111.9 ± 3.1	111.5 ± 3.6	93.0 ± 2.0***	122.6 ± 2.1^#^	91.1 ± 2.8*	106.5 ± 2.4	106.8 ± 2.4	110.9 ± 2.5	96.5 ± 3.0***	104.2 ± 2.5^#^	95.4 ± 2.5*
CHO (mmol/L)	–	–	–	–	–	–	3.9 ± 0.16	3.9 ± 0.14	3.9 ± 0.12	5.9 ± 0.62***	4.1 ± 0.19^#^	6.9 ± 0.60*
TG (mmol/L)	–	–	–	–	–	–	3.3 ± 0.37	3.2 ± 0.12	3.0 ± 0.13	6.1 ± 0.61***	4.6 ± 0.21**^#^	6.9 ± 0.74*
HDL (mmol/L)	–	–	–	–	–	–	2.8 ± 0.07	2.8 ± 0.07	2.9 ± 0.07	3.4 ± 0.19	2.9 ± 0.10	3.4 ± 0.24
LDL (mmol/L)	–	–	–	–	–	–	0.81 ± 0.05	0.81 ± 0.06	0.81 ± 0.03	1.39 ± 0.15***^#^	0.76 ± 0.03^#^	2.04 ± 0.25*

Data are expressed as mean ± SE, *n* = 7 in all groups

*P < 0.05 vs. CON group; **P < 0.05 vs. TUDCA group; ***P < 0.05 vs. PBA group; ^#^*P* < 0.05 vs. DM group

*BW* body weight, *Glu* serum glucose, *GSP* glycosylated serum protein, *BUN* blood urea nitrogen, *Cre* serum creatinine, *Mg* serum magnesium, *Ca* serum calcium, *CHO* total cholesterol, *TG* triglyceride, *HDL* high-density lipoprotein cholesterol, *LDL* low-density lipoprotein

At 2 months, the DM mice had significantly higher GSP (475.4 ± 19.2 vs. 259.6 ± 17.9 μmol/L; *P* < 0.05) and BUN (16.8 ± 0.59 vs. 11.3 ± 0.39 mmol/L; *P* < 0.05) levels than the control mice, but there were no significant differences between GSP and BUN levels in PBA + DM or TUDCA + DM mice compared to DM mice.

At 2 months, DM mice had significantly higher Cre (20.5 ± 2.30 vs. 12.7 ± 0.28 mmol/L; *P* < 0.05) levels than control mice, there was no significant difference between Cre levels in TUDCA + DM mice compared to DM mice, but PBA + DM had significantly lower Cre (13.3 ± 0.94 vs. 20.5 ± 2.30 mmol/L; *P* < 0.05) levels than the DM mice, with values similar to control levels.

At 2 months, DM mice had significantly higher CHO (6.9 ± 0.60 vs. 3.9 ± 0.16 mmol/L; *P* < 0.05) and TG (6.9 ± 0.74 vs. 3.3 ± 0.37 mmol/L; *P* < 0.05) levels than control mice, there were no significant differences between CHO and TG levels in PBA + DM mice compared to DM mice, but TUDCA + DM mice had significantly lower CHO (4.1 ± 0.19 mmol/L; *P* < 0.05 vs. DM mice) and TG (4.6 ± 0.21 mmol/L; *P* < 0.05 vs. DM mice) levels than the DM mice, with some values approaching control levels.

At 2 months, the DM mice had significantly higher LDL (2.04 ± 0.25 vs. 0.81 ± 0.05 mmol/L; *P* < 0.05) levels than control mice, but PBA + DM or TUDCA + DM had significantly lower LDL (PBA + DM, 1.39 ± 0.15 mmol/L; TUDCA + DM 0.76 ± 0.03 mmol/L; *P* < 0.05 vs. DM mice) levels than the DM mice, with some values approaching control levels.

### Mg^2+^ and Ca^2+^ Levels in the Heart, Liver, Kidney, Muscle, Spleen, and Serum

**Fig. 1:**
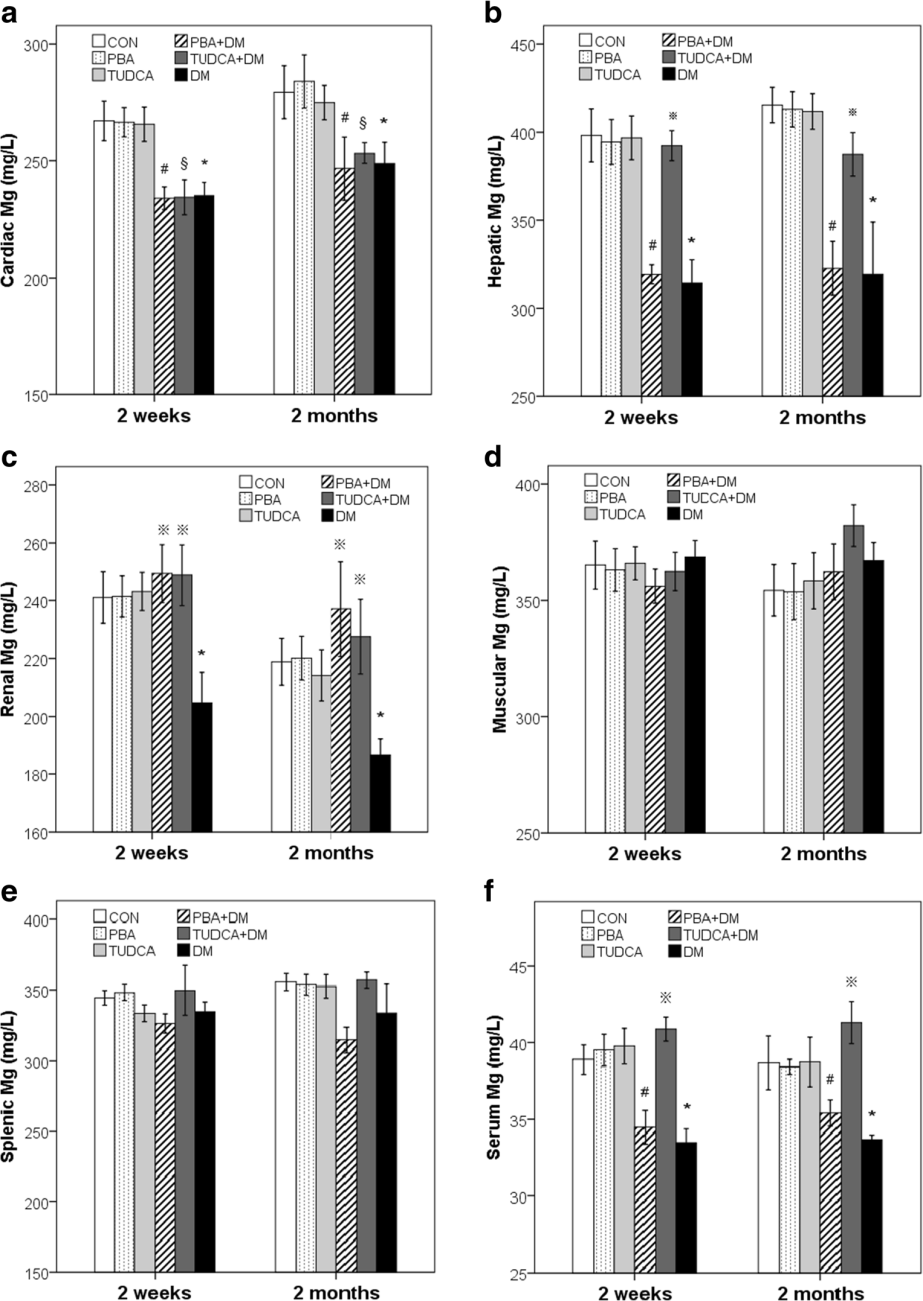
Mg^2+^ levels in the heart, liver, kidney, muscle, spleen, and serum of the DM mice. Mg^2+^ levels in the heart (**a**), liver (**b**), kidney (**c**), muscle (**d**), spleen (**e**), and serum (**f**) after 2 weeks and 2 months of treatment with PBA or TUDCA. Data are presented as mean ± SE, *n* = 7 in all groups. *CON* non-diabetic control mice group, *DM* diabetic mice group, *PBA + DM* group of diabetic mice treated with PBA, *TUDCA + DM* group of diabetic mice treated with TUDCA, *PBA* group of non-diabetic control mice treated with PBA, *TUDCA* group of non-diabetic control mice treated with TUDCA

Mg^2+^ levels in the heart, liver, kidney, muscle, spleen, and serum are summarized in Table 1 and Fig. 1. At 2 weeks and 2 months, the DM, PBA + DM, and TUDCA + DM mice had significantly lower cardiac Mg^2+^ (DM, 235.1 ± 14.9 mg/L; PBA + DM, 234.0 ± 12.7 mg/L; TUDCA + DM, 234.4 ± 19.6 mg/L vs. 267.4 ± 22.3 mg/L in 2 weeks; DM, 248.8 ± 25.1 mg/L; PBA + DM, 246.8 ± 36.2 mg/L; TUDCA + DM, 253.5 ± 12.2 mg/L vs. 279.5 ± 29.7 mg/L in 2 months; *P* < 0.05) levels compared to the control mice. At 2 weeks and 2 months, the DM mice had significantly lower hepatic Mg^2+^ (2 weeks, 314.4 ± 35.0 vs. 398.2 ± 39.8 mg/L; 2 months, 319.4 ± 77.9 vs. 415.5 ± 26.7 mg/L; *P* < 0.05) levels compared to the control mice; the TUDCA + DM mice had significantly higher hepatic Mg^2+^ (2 weeks, 392.4 ± 22.6 mg/L; 2 months, 387.4 ± 32.7 mg/L; *P* < 0.05 vs. DM mice) levels than the DM mice, with values similar to control levels; and there was no significant difference between hepatic Mg^2+^ levels in the PBA + DM mice compared to the DM mice. At 2 weeks and 2 months, the DM mice had significantly lower renal Mg^2+^ (2 weeks, 204.7 ± 27.8 vs. 241.0 ± 23.5 mg/L; 2 months, 186.9 ± 14.6 vs. 218.9 ± 21.4 mg/L; *P* < 0.05) levels compared to the control mice, and the TUDCA + DM mice and PBA + DM mice had significantly higher renal Mg^2+^ (TUDCA + DM, 248.8 ± 27.9 mg/L; PBA + DM, 249.3 ± 26.6 mg/L in 2 weeks; TUDCA + DM, 227.5 ± 34.0 mg/L; PBA + DM, 237.1 ± 43.5 mg/L in 2 months; *P* < 0.05 vs. DM mice) levels than the DM mice, with values similar to control levels. At each time point, there were no significant differences in splenic or muscular Mg^2+^ levels in the DM, PBA + DM, TUDCA + DM, and control mice. At 2 weeks and 2 months, the DM mice had significantly lower serum Mg^2+^ (2 weeks, 33.5 ± 0.89 vs. 38.9 ± 0.95 mg/L; 2 months, 33.7 ± 0.30 vs. 38.7 ± 1.75 mg/L; *P* < 0.05) levels compared to the control mice, the TUDCA + DM mice had significantly higher serum Mg^2+^ (2 weeks, 40.9 ± 0.79 mg/L; 2 months, 41.3 ± 1.39 mg/L; *P* < 0.05 vs. DM mice) levels than the DM mice, with values similar to control levels; and there were no significant differences between serum Mg^2+^ levels in the PBA + DM mice compared to the DM mice.

**Fig. 2:**
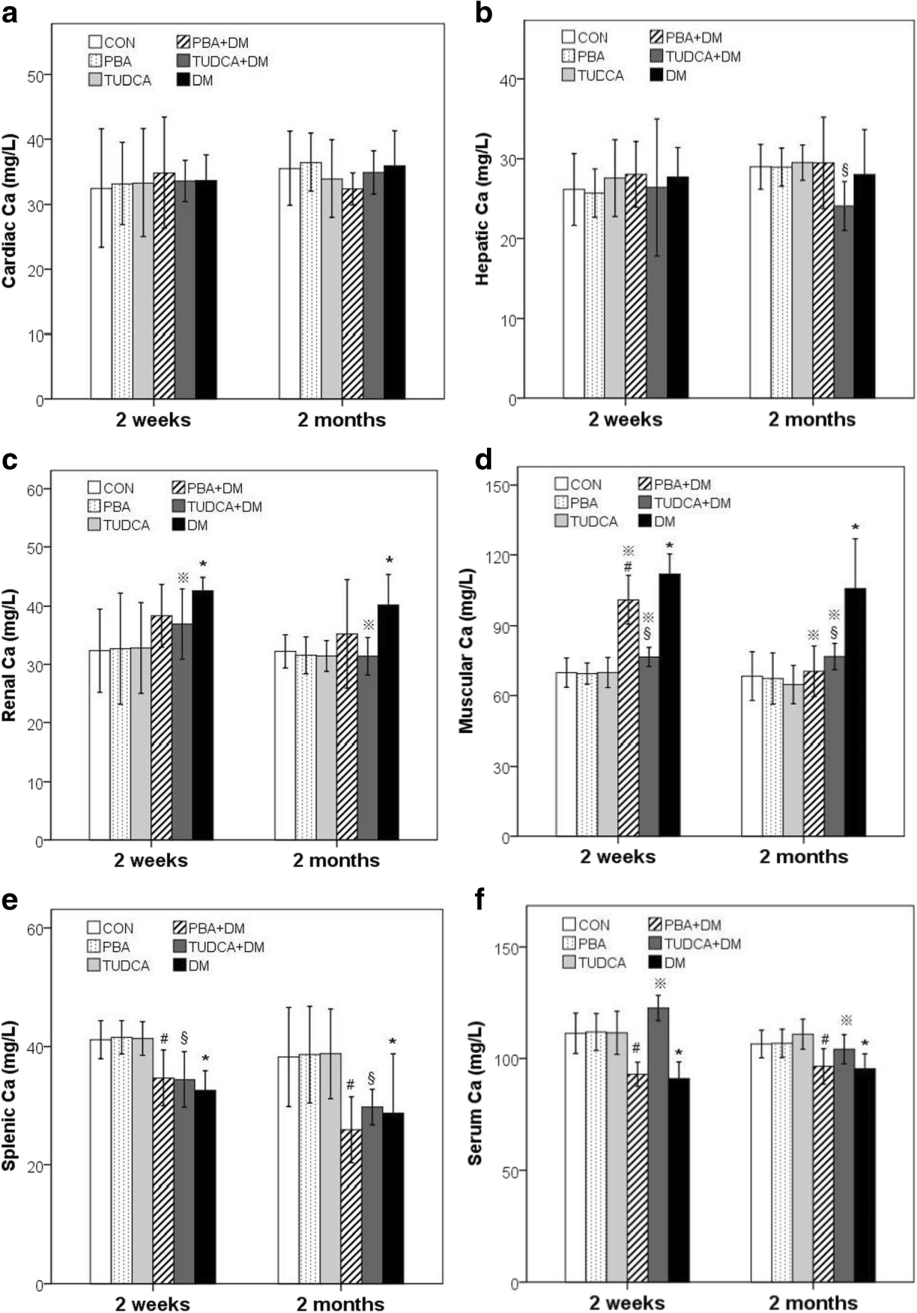
Ca^2+^ levels in the heart, liver, kidney, muscle, spleen, and serum of the DM mice. Ca^2+^ levels in the heart (**a**), liver (**b**), kidney (**c**), muscle (**d**), spleen (**e**), and serum (**f**) after 2 weeks and 2 months of treatment with PBA or TUDCA. Data are presented as mean ± SE, *n* = 7 in all groups

Ca^2+^ levels in the heart, liver, kidney, muscle, spleen, and serum are summarized in Table 1 and Fig. 2. At each time point, there were no significant differences in cardiac Ca^2+^ levels in the DM, PBA + DM, TUDCA + DM, and control mice. At 2 weeks, there were no significant differences in hepatic Ca^2+^ levels in the DM, PBA + DM, TUDCA + DM, and control mice, but at 2 months, hepatic Ca^2+^ (24.1 ± 3.1 vs. 29.0 ± 2.8 mg/L; *P* < 0.05) levels in the TUDCA + DM mice were significantly lower than the control mice. At 2 weeks and 2 months, renal Ca^2+^ (2 weeks, 42.6 ± 2.3 vs. 32.3 ± 7.2 mg/L; 2 months, 40.2 ± 5.2 vs. 32.2 ± 2.9 mg/L; *P* < 0.05) levels in the DM mice were significantly higher than the control mice, and renal Ca^2+^ (2 weeks, 36.9 ± 6.0 mg/L; 2 months, 31.4 ± 3.2 mg/L; *P* < 0.05 vs. DM mice) levels in the TUDCA + DM were significantly lower than the DM mice, with values similar to control levels. At 2 weeks and 2 months, muscular Ca^2+^ (2 weeks, 112.1 ± 8.2 vs. 69.9 ± 6.3 mg/L; 2 months, 106.0 ± 20.9 vs. 68.4 ± 10.4 mg/L; *P* < 0.05) levels in the DM mice were significantly higher than the control mice, and muscular Ca^2+^ (PBA + DM, 101.1 ± 10.4 mg/L; TUDCA + DM, 76.6 ± 4.1 mg/L in 2 weeks; PBA + DM, 70.4 ± 10.9 mg/L; TUDCA + DM, 76.8 ± 5.7 mg/L in 2 months) levels in the PBA + DM and TUDCA + DM mice were significantly lower than the DM mice, although most values did not return to control levels. At each time point, the DM, PBA + DM, and TUDCA + DM mice had significantly lower splenic Ca^2+^ (DM, 32.6 ± 3.3 mg/L; PBA + DM, 34.7 ± 4.7 mg/L; TUDCA + DM, 34.5 ± 4.7 mg/L vs. 41.2 ± 3.2 mg/L in 2 weeks; DM, 28.8 ± 10.0 mg/L; PBA + DM, 25.9 ± 5.6 mg/L; TUDCA + DM, 29.8 ± 3.0 mg/L vs. 38.3 ± 8.4 mg/L in 2 months; *P* < 0.05) levels than the control mice. At 2 weeks and 2 months, serum Ca^2+^ (2 weeks, 91.1 ± 2.8 vs. 111.3 ± 3.4 mg/L; 2 months, 95.4 ± 2.5 vs. 106.5 ± 2.4 mg/L; *P* < 0.05) levels in the DM mice were significantly lower than the control mice; serum Ca^2+^ (2 weeks, 122.6 ± 2.1 mg/L; 2 months, 104.2 ± 2.5 mg/L; *P* < 0.05) levels in the TUDCA + DM mice were significantly higher than the DM mice, with values similar to control levels; and there were no significant differences between serum Ca^2+^ levels in PBA + DM mice compared to DM mice.

### Correlation of Mg^2+^ and Ca^2+^ Levels in the Heart, Liver, Kidney, Muscle, Spleen, and Serum

**Fig. 3:**
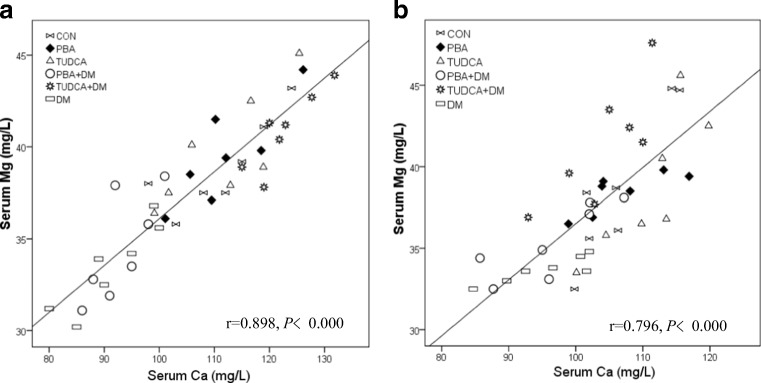
Correlation between serum Mg^2+^ and Ca^2+^ levels in the DM mice. Correlations between serum Mg^2+^ and serum Ca^2+^ levels after 2 weeks (*r* = 0.898, *P* < 0.001, **a**) and 2 months (*r* = 0.796, *P* < 0.001, **b**) of treatment with PBA or TUDCA

Correlations between Mg^2+^ and Ca^2+^ levels in the heart, liver, kidney, muscle, spleen, and serum of the DM mice after 2 weeks and 2 months of treatment with PBA or TUDCA are summarized in Tables 2 and 3 Fig. 3. Mg^2+^ levels were positively correlated with Ca^2+^ levels in the heart, liver, kidney, muscle, spleen, and serum in all groups of mice at 2 weeks and 2 months, except in the liver of the PBA + DM or TUDCA + DM mice at both time points, and the heart of the TUDCA + DM mice at 2 weeks.

**Table 2:** Correlations between Mg^2+^ and Ca^2+^ levels in the heart, liver, kidney, muscle, spleen, and serum of diabetic mice after 2 weeks of treatment with PBA or TUDCA

	Mg											
	CON		PBA		TUDCA		PBA + DM		TUDCA+DM		DM	
	*r*	*P*	*r*	*P*	*r*	*P*	*r*	*P*	*r*	*P*	*r*	*P*
H-Ca	0.937	0.002*	0.893	0.007*	0.857	0.014*	0.857	0.014*	0.214	0.645	0.964	0.000*
L-Ca	0.857	0.014*	0.786	0.036*	0.929	0.003*	0.036	0.939	0.432	0.333	0.883	0.008*
K-Ca	0.786	0.036*	0.821	0.023*	0.857	0.014*	0.857	0.014*	0.893	0.007*	0.857	0.014*
M-Ca	0.883	0.008*	0.857	0.014*	0.857	0.014*	0.821	0.023*	0.857	0.014*	0.857	0.014*
P-Ca	0.821	0.023*	0.929	0.003*	0.929	0.003*	0.857	0.014*	0.821	0.023*	0.857	0.014*
S-Ca	0.786	0.036*	0.857	0.014*	0.929	0.003*	0.821	0.023*	0.821	0.023*	0.847	0.016*

*H-Ca* calcium level in the heart, *L-Ca* calcium level in the liver, *K-Ca* calcium level in the kidney, *M-Ca* calcium level in the muscle, *P-Ca* calcium level in the spleen, *S-Ca* calcium level in serum

*P < 0.05 for the correlation

**Table 3:** Correlations between Mg^2+^ and Ca^2+^ levels in the heart, liver, kidney, muscle, spleen, and serum of diabetic mice after 2 months of treatment with PBA or TUDCA

	Mg											
	CON		PBA		TUDCA		PBA + DM		TUDCA+DM		DM	
	*r*	*P*	*r*	*P*	*r*	*P*	*r*	*P*	*r*	*P*	*r*	*P*
H-Ca	0.893	0.007*	0.857	0.014*	0.929	0.003*	0.929	0.003*	0.857	0.014*	0.873	0.010*
L-Ca	0.929	0.003*	0.821	0.023*	0.821	0.023*	0.357	0.432	0.429	0.337	0.893	0.007*
K-Ca	0.857	0.014*	0.893	0.007*	0.893	0.007*	0.893	0.007*	0.821	0.023*	0.857	0.014*
M-Ca	0.929	0.003*	0.786	0.036*	0.821	0.023*	0.857	0.014*	0.937	0.002*	0.901	0.006*
P-Ca	0.821	0.023*	0.857	0.014*	0.857	0.014*	0.857	0.014*	0.857	0.014*	0.929	0.003*
S-Ca	0.775	0.041*	0.857	0.014*	0.821	0.023*	0.857	0.014*	0.857	0.014*	0.893	0.007*

*H-Ca* calcium level in the heart, *L-Ca* calcium level in the liver, *K-Ca* calcium level in the kidney, *M-Ca* calcium level in the muscle, *P-Ca* calcium level in the spleen, *S-Ca* calcium level in serum

*P < 0.05 for the correlation

## Discussion

This study established and characterized a mouse model of T1D and investigated the effect of TUDCA and PBA on Mg^2+^and Ca^2+^ metabolism in these mice. Findings showed T1D mice had decreased body weight and increased blood glucose at 2 weeks and 2 months after chaperone or vehicle administration was initiated, and GSP, BUN, Cre, CHO, TG, and LDL levels were significantly increased in T1D mice at 2 months. PBA or TUDCA administration attenuated the changes in body weight and blood glucose in T1D mice at 2 weeks and 2 months. PBA administration attenuated the changes in Cre levels, and TUDCA attenuated the changes in CHO, TG, and LDL levels at 2 months.

In addition, the present study comprehensively evaluates Mg^2+^ and Ca^2+^ levels in the heart, liver, kidney, muscle, spleen, and serum of T1D mice. Findings showed T1D mice experienced alterations in Mg^2+^ and Ca^2+^ homeostasis; Mg^2+^ levels were significantly decreased in the heart, liver, kidney, and serum of T1D mice compared to controls. Ca^2+^ levels were significantly decreased in the spleen and serum of T1D mice compared to controls, but significantly increased in the kidney and muscle.

Mg^2+^ and Ca^2+^ are important micronutrients that are involved in several pathophysiological mechanisms. A deficiency in Mg^2+^ in T1D may result from insufficient dietary intake, decreased absorption in the gastrointestinal tract [17], altered reabsorption in the kidney due to hyperglycemia and hypersthenuria [18], and/or redistribution in the body due to metabolic and/or acid-base disorders [18]. Diabetes is characterized by a negative Mg^2+^ balance, which may manifest as hypocalcaemia, hypokalemia, and cardiac and neurological dysfunction [19]. Ca^2+^ homeostasis may be disrupted by obesity, which influences cellular Ca^2+^ flux between the cytosol and organelles [6]. Ca^2+^ regulates insulin and glucagon secretion and the control of blood glucose [6]. Ca^2+^ plays an important role in muscle contraction; therefore, altered cellular Ca^2+^ levels can impact the heart and skeletal muscle [6]. Further, Ca^2+^ is essential for adequate hepatic glucose production, lipogenesis, inflammation, and other critical metabolic processes [6]. Findings from the present study suggest a potential role for therapeutic strategies targeted at maintaining Mg^2+^ and Ca^2+^ homeostasis in tissues and serum in the management of diabetes.

The present study assessed the relationship between Mg^2+^ and Ca^2+^ levels in tissues and serum of T1D mice. Findings showed that Mg^2+^ levels in the heart, liver, kidney, muscle, spleen, and serum were positively correlated with Ca^2+^ levels in T1D and control mice at 2 weeks and 2 months after chaperone or vehicle administration was initiated, but not in T1D mice administered PBA or TUDCA. These findings are in accordance with our previous study, in which serum Mg^2+^ levels were positively correlated with Ca^2+^ levels in individuals with T2D [2], and urinary Mg^2+^ levels were positively correlated with urinary Ca^2+^ in controls and individuals with impaired glucose tolerance or T2D [2]. In other studies, there were significant positive correlations between Mg^2+^ and Ca^2+^ urinary excretion rates, clearance, and excretion fractions in individuals with diabetes in Nigeria [20], and serum Mg^2+^ and Ca^2+^ levels and the interaction between serum Mg^2+^ and Ca^2+^ levels (Mg^2+^ × Ca^2+^) had significant negative correlations with eGFR [17] in individuals with T2D; (Mg^2+^ × Ca^2+^) had the strongest correlation with eGFR [17]. Taken together, these data imply positive associations and possible regulatory effects between tissue and serum Mg^2+^ and Ca^2+^ levels in healthy individuals and those with diabetes. Further animal experiments and clinical investigations are required to elucidate the underlying mechanisms and relevance of these observations.

Chemical or pharmaceutical chaperones, including PBA and TUDCA, are a group of low molecular weight compounds that can stabilize protein conformation, improve the folding capacity of the ER, and facilitate the trafficking of mutant proteins [21]. TUDCA is a taurine-conjugated bile acid that has been safely used in Traditional Chinese Medicine (dried bile from adult black bears) and in the Western World to treat biliary and liver diseases [22]. PBA was approved by the US Food and Drug Administration as an ammonia scavenger for the treatment of urea-cycle disorders and has been investigated in clinical trials in patients with thalassemia and cystic fibrosis [15]. Evidence suggests that PBA and TUDCA can confer antiapoptotic, anti-inflammatory, antioxidant and immunomodulation effects [22, 23], enhance ER adaptability, and prevent insulin resistance [21, 22].

In the present study, PBA restored renal Mg^2+^ and muscular Ca^2+^ to control levels in the DM mice 2 weeks and 2 months after chaperone or vehicle administration was initiated, and TUDCA restored hepatic, renal, and serum Mg^2+^ and renal and serum Ca^2+^ to control levels. This study confirmed that molecular chaperones have a role in the regulation of micronutrients in a mouse model of T1D.

These findings are in accordance with several previous reports. In a mouse model of pulmonary hypertension, PBA reversed the hypoxia-induced alterations in mitochondrial Ca^2+^ levels and the activity of Ca^2+^-sensitive mitochondrial enzymes in pulmonary artery smooth muscle, thereby preventing and reversing pulmonary hypertension [24]. In Leishmania parasites, PBA inhibited Ca^2+^-induced mitochondrial toxicity and inhibited DNA degradation, phosphatidylserine exposure, and apoptosis [25]. In bile duct ligated-rats that had undergone vagotomy, TUDCA prevented Ca^2+^-induced apoptosis and the loss of proliferative and functional responses [26]. In obese mice, TUDCA regulated intracellular calmodulin and prevented damage to the myocardium [22].

Interestingly, in the present study, the effect of DM, PBA, and TUDCA on Mg^2+^ and Ca^2+^ levels in T1D mice varied according to the tissue tested. This may because the number of mitochondria, ER, and ion exchange pumps, and/or the concentrations of micronutrients vary across tissues.

## Conclusions

In conclusion, to the author’s knowledge, this is first study to systematically investigate the effects of PBA and TUDCA on Mg^2+^ and Ca^2+^ metabolism in tissues and serum in a mouse model of T1D. Findings showed that (1) Mg^2+^ levels in the heart, liver, kidney, and serum and Ca^2+^ levels in the spleen and serum of T1D mice were significantly decreased compared to controls, while Ca^2+^ levels in the kidney and muscle were significantly increased; (2) Mg^2+^ and Ca^2+^ levels in the heart, liver, kidney, muscle, spleen and serum were positively correlated in control and T1D mice at 2 weeks and 2 months after chaperone or vehicle administration was initiated; (3) PBA restored renal Mg^2+^ levels to normal values at 2 weeks and 2 months after chaperone or vehicle administration was initiated and muscular Ca^2+^ levels to normal values at 2 months, TUDCA restored hepatic, renal, and serum Mg^2+^ levels and renal and serum Ca^2+^ levels to normal values at 2 weeks and 2 months after chaperone or vehicle administration was initiated. Further research is required to investigate the underlying mechanisms by which chaperones regulate micronutrients.
